# The role of public health networks in strengthening public health systems: the case of EMPHNET in the Eastern Mediterranean region

**DOI:** 10.3389/fpubh.2025.1654089

**Published:** 2025-08-29

**Authors:** Yousef Khader, Dana Shalabi, Leen Daoud, Deema Al Bakri, Rana AlHamawi, Randa K. Saad, Sayed Himatt, Zeina Abdel Majeed, Ruba Kamal Alsouri, Nada Ahmed, Rabi Assaf, Nadine Haddad, Sardar Parwiz, Majd A. Alsoukhni, Haitham Bashier, Magid Al-Gunaid, Mohannad Al Nsour

**Affiliations:** ^1^Department of Public Health, Jordan University of Science and Technology, Irbid, Jordan; ^2^Media, Communications, and Networking, EMPHNET, Amman, Jordan; ^3^Polio and Routine Immunization, EMPHNET, Amman, Jordan; ^4^Disease Control and Prevention, EMPHNET, Amman, Jordan; ^5^Public Health Programs, Global Health Development, Eastern Mediterranean Public Health Network, Amman, Jordan; ^6^Department of Research and Policy, Global Health Development, Eastern Mediterranean Public Health Network, Amman, Jordan; ^7^Public Health Emergency Management Center, EMPHNET, Amman, Jordan; ^8^Workforce Capacity Team, EMPHNET, Amman, Jordan; ^9^Public Health Programs, EMPHNET, Amman, Jordan; ^10^The Eastern Mediterranean Public Health Network, Amman, Jordan; ^11^Polio and immunization Unite, Public Health Department, The Eastern Mediterranean Public Health Network, Amman, Jordan

**Keywords:** public health networks, EMPHNET, Eastern Mediterranean region, disease surveillance, immunization, workforce development, One Health, health systems strengthening

## Abstract

The Eastern Mediterranean Region (EMR) faces a range of complex public health challenges, including endemic diseases, recurrent outbreaks, and weak health systems exacerbated by political instability and humanitarian crises. Public health networks play a pivotal role in addressing these challenges by fostering collaboration, enhancing workforce capacity, and strengthening disease prevention and response systems. This manuscript examines the contributions of the Eastern Mediterranean Public Health Network (EMPHNET) as a model for leveraging public health networks to improve health systems and outcomes in the EMR. EMPHNET operates across multiple public health domains, including disease surveillance, routine immunization, polio eradication, emergency management, non-communicable diseases, neglected tropical disease (NTDs), and One Health initiatives. Through its strategic partnerships with ministries of health, international organizations, and academic institutions, EMPHNET has strengthened epidemiological capacity, supported mass vaccination campaigns, and advanced research and policy development. Notable initiatives include enhancing syndromic surveillance and implementing laboratory-based brucellosis surveillance. In addition to infectious disease control, EMPHNET has made significant strides in maternal and child health, workforce development, and digital health innovations. It has supported national immunization strategies, developed digital health tools for real-time surveillance, and provided capacity-building programs for frontline healthcare workers. Furthermore, EMPHNET's contributions to biosecurity, antimicrobial resistance, and NTDs underscore its commitment to addressing both regional and global health threats. In conclusion, EMPHNET has emerged as a leading organization in strengthening public health systems in the EMR. Its work highlights the critical role of public health networks in building resilience, enhancing emergency preparedness, and improving population health outcomes.

## Introduction

The Eastern Mediterranean Region (EMR), as defined by the World Health Organization, comprises 22 countries and the occupied Palestinian territory. These include Afghanistan, Bahrain, Djibouti, Egypt, the Islamic Republic of Iran, Iraq, Jordan, Kuwait, Lebanon, Libya, Morocco, Oman, Pakistan, Palestine Qatar, Saudi Arabia, Somalia, Sudan, the Syrian Arab Republic, Tunisia, the United Arab Emirates, and Yemen. The EMR faces significant public health challenges, including endemic diseases, recurring outbreaks, and the emergence of novel pathogens. Infectious diseases caused by emerging, re-emerging, and high-threat pathogens continue to cause increased morbidity and mortality in the region, particularly in countries undergoing humanitarian crises (1). The EMR remains the only World Health Organization (WHO) region worldwide that is yet to be certified polio-free, as wild poliovirus transmission remains endemic in Afghanistan and Pakistan (2). Furthermore, outbreaks of circulating vaccine-derived poliovirus (cVDPV) still occur, particularly in countries with sub-optimal vaccination coverage such as Somalia, Yemen, and Sudan, or low immunity pockets such as Egypt, and recently in the Gaza Strip (3). Many countries in the region have fragile health systems, struggling to provide proper and adequate surveillance, early detection, and response to disease outbreaks, coupled with a shortage of trained public health professionals and a lack of resources. Limited access to vaccines and some medications is a challenge in some areas of the region. These challenges are compounded by weak health systems, political instability, ongoing conflicts, economic disparities, and environmental conditions (4).

Moreover, Neglected Tropical Diseases (NTDs) represent a critical public health challenge in the EMR, particularly in areas affected by poverty and limited access to healthcare. In 2022, the EMR accounted for 4.6% of the global population requiring interventions against NTDs, translating to a significant burden across the region (5). Despite progress in some countries, such as Iraq, which eliminated trachoma as a public health problem, and Yemen, which achieved the elimination of lymphatic filariasis, the overall burden remains considerable.

The region is also home to some of the worst health and humanitarian crises in the world (6). Twelve of the 22 member states and territories of the EMR are directly or indirectly impacted by acute or protracted humanitarian emergencies contributing to fragile health systems and high numbers of internally displaced persons and refugees, often with limited access to basic healthcare services and environmental infrastructure (7).

Furthermore, the EMR faces the highest rate of premature mortality due to the four main non-communicable diseases (NCDs)—cardiovascular diseases, cancer, diabetes, and chronic respiratory diseases. Despite global progress, the EMR's reduction in NCD mortality lags behind other regions (8), emphasizing the urgent need for targeted interventions. Another critical gap in the region is the shortage of competent public health professionals, particularly in specialized areas such as epidemiology, emergency preparedness, and disease surveillance.

Global health challenges demand coordinated responses that transcend geographic and administrative boundaries. Public health networks have emerged as vital platforms for collaboration, bringing together diverse stakeholders such as governments, healthcare providers, academic institutions, and non-governmental organizations (NGOs). Key contributions of public health networks include coordinating responses to health emergencies, enhancing workforce capacity, promoting health equity, and advancing research and evidence-based practices. Their impact extends to strengthening health systems, improving population health, and driving innovation in disease prevention, control, and health policy.

This manuscript examines the Eastern Mediterranean Public Health Network (EMPHNET) as a case study to explore the multifaceted contributions of public health networks in the EMR. We used a qualitative case study approach, conducting a desk review of EMPHNET's internal reports, project briefs, website content, evaluations, and relevant external publications to assess EMPHNET's impact in the EMR. Data were thematically organized by EMPHNET's major work domains and synthesized to highlight contributions and outcomes, supported by its own monitoring and evaluation results. The approach is descriptive, drawing on triangulated evidence from multiple sources to illustrate EMPHNET's role in strengthening health systems across the region.

## EMPHNET: a model of public health network

The EMPHNET is a public health nonprofit organization established to support better health in the EMR and beyond. EMPHNET's core aim is to improve public health and well-being in the EMR and beyond. Established in 2009 as a non-profit public health network, EMPHNET's vision is better health for people in the EMR, and its goals can be summarized as follows: to prevent and control diseases by strengthening the public health workforce, conducting operational research, improving public health programs, and promoting knowledge exchange and networking. In practice, this means EMPHNET works to build capacity in field epidemiology, support disease surveillance and immunization initiatives, advance public health programs and research, and facilitate collaboration among stakeholders for stronger health systems. To achieve its goals, it works with ministries of health and public health agencies, universities, community organizations, research institutes, the private sector, NGOs, international organizations, and United Nations agencies, among others.

EMPHNET tracks a defined set of key performance indicators, combining “lagging” outcome measures and “leading” output measures, to monitor progress toward its strategic goals. These include the number of Field Epidemiology Training Programs (FETPs) established or supported, public health professionals trained, coverage of interventions, geographic and programmatic reach, and measurable health outcomes. Indicators are tied to strategic objectives with set targets, monitored quarterly, and reviewed regularly to ensure accountability and assess impact on public health in the EMR.

## Disease prevention and control

EMPHNET has successfully contributed to the enhancement of the region's ability to prevent, identify, and respond to outbreaks and health emergencies by tackling a variety of complex health issues, from infectious diseases to emerging global threats. Through focusing on innovative solutions and investing in sustainable capacity-building of local experience, EMPHNET has fostered multisectoral collaboration and bridged gaps in health systems. EMPHNET has made notable strides in strengthening disease surveillance and public health capacities in the EMR. Its collaboration with the Iraqi Ministry of Health since 2014 has led to enhanced syndromic surveillance during the Arba'een pilgrimage, one of the world's largest annual gatherings. This initiative included upgrading surveillance tools, customizing mobile data collection software, and training health staff to manage real-time data from 187 health facilities along the pilgrimage route. These efforts have significantly improved Iraq's capacity to detect and respond to infectious disease outbreaks during mass gatherings (9).

From 2018 to 2022, EMPHNET collaborated with the Centers for Disease Control and Prevention (CDC) on a laboratory-based surveillance project for human and animal brucellosis in Jordan, funded by the Defense Threat Reduction Agency. This project improved surveillance tools, enhanced laboratory capacities, distributed kits, and trained over 300 professionals, including laboratory technicians, health clinicians, and veterinarians. The initiative applied a One Health approach to zoonotic illnesses and created standardized protocols for specimen transport and storage, significantly contributing to reducing the brucellosis burden in Jordan (10).

To address gaps in epidemiological data for meningitis in the region, EMPHNET launched the Meningitis and Septicemia Mapping Network (MenMap). This initiative operates in Egypt, Iraq, and Jordan, aiming to enhance the diagnosis and surveillance of invasive bacterial diseases, including *Neisseria meningitidis, Streptococcus pneumoniae, and Haemophilus influenzae*. By implementing real-time Polymerase Chain Reaction techniques and building a robust laboratory network, MenMap seeks to advance vaccine-preventable disease diagnostics, guide public health policy, and improve clinical management of bacterial meningitis and septicemia (11).

EMPHNET also plays an active role in combating antimicrobial resistance (AMR) in the EMR through projects focusing on AMR surveillance in farms, infection prevention in healthcare settings, and training to reduce antibiotic misuse. By collaborating with global coalitions like the AMR Multi-Stakeholder Partnership Platform, EMPHNET has enhanced AMR response capacities and fostered knowledge sharing across sectors.

## Routine immunization and polio eradication

Over the past years, EMPHNET has supported national public health initiatives in several domains on routine vaccination and polio eradication. Through long-standing alliances with regional and international health stakeholders as well as health ministries, EMPHNET has amassed immense expertise in enhancing the capacity of health ministries to boost vaccine demand, fortify public-private partnerships, and strengthen the immunization system. EMPHNET provided technical and logistical support aiming to build the capacity in immunization microplanning and strengthening supportive supervision of public health workers and vaccinators across Afghanistan, Egypt, Iraq, Jordan, Morocco, Pakistan, Sudan, Somalia, and Yemen. Other supporting activities included developing training curricula on best practices for routine immunization microplanning, creating health facility-based microplans, and providing technical support for their validation and implementation.

EMPHNET has extensive experience in advocating for community-based initiatives as a driving force for the elimination, eradication, and control of vaccine-preventable diseases (VPDs). It has carefully tailored contextual, community-centered approaches in Pakistan, Sudan, Yemen, Somalia, and Afghanistan to increase vaccination demand while addressing gender-based inequities and vaccination barriers. Recently, in response to the confirmation of cVDPV type 2 in environmental samples and a polio case in the Gaza Strip, EMPHNET has been actively involved in providing technical and logistical support. Working alongside local and regional partners, it contributes to developing microplanning strategies and field response activities during the implementation phase.

To raise awareness of the value of vaccinations and foster confidence in immunization programs in Afghanistan, EMPHNET developed and disseminated context-based Information, Education, and Communication materials and voice messages, and engaged community and religious leaders in building the capacity of frontline health workers on interpersonal communication in countries such as Iraq and Afghanistan. Currently, EMPHNET is supporting updating the communication strategy in Sudan to be aligned with the emergency context and new policy on life-course vaccination and enhance integration, including immunization and communication strategy during emergencies, with the full engagement of relevant stakeholders.

EMPHNET's extensive experience in strengthening Acute Flaccid Paralysis (AFP) and other VPD surveillance includes efforts to reinforce AFP surveillance indicators, as demonstrated in Morocco. In Iraq, EMPHNET supported the Ministry of Health in conducting an internal independent review of AFP surveillance to assess progress in implementing province-specific recommendations. In Egypt, EMPHNET leveraged the existing polio eradication infrastructure to support broader public health goals. Through the implementation of the polio legacy project, it identified areas with low population immunity for measles in selected governorates. This project provided critical insights into reaching high-risk populations to enhance both surveillance and routine immunization, ultimately helping to overcome challenges in VPD surveillance and routine immunization activities.

To prevent, detect, and respond to VPD outbreaks according to country-specific needs, EMPHNET worked closely with MOHs and country partners to support WPV and cVDPV outbreak response and preparedness in Afghanistan, Somalia, and Yemen. As a response to the polio outbreak in the Gaza Strip, EMPHNET is supporting community mobilizers in all of Gaza to raise awareness about the risks of polio and promote polio vaccine demand and uptake during the two rounds of the campaign, in close support of local health authorities and regional partners.

Driven by its technical expertise and intense experience in immunization policies and strengthening health systems for more efficient and equitable vaccine delivery, EMPHNET supports the ministries of health in the region in updating their national immunization policies, whether through the introduction of novel vaccines or through revising the immunization calendar, in the light of occurring local and regional public health issues. EMPHNET is prioritizing the strengthening of National Immunization Technical Advisory Groups (NITAGs) as a key national technical resource for vaccination and immunization guidance. This effort is being implemented through close coordination with the WHO Headquarters (WHO-HQ), the WHO Eastern Mediterranean Regional Office (WHO-EMRO), the United States CDC, EMPHNET, the Task Force for Global Health (TFGH), and the Network for Immunization Safety and Health (NISH).

As part of EMPHNET's continuous commitment to the control of VPDs in the region, EMPHNET is collaborating on a new project entitled “The Way Forward for Pertussis Control in the EMR: Focus on Maternal Immunization”. Specifically, this project aimed to create a regional network of experts in Pertussis and maternal immunization policies in EMR to share successful international and local experiences in pertussis control, understand the drivers and barriers for Tdap introduction in pregnancy, raise the value of Tetanus, Diphtheria, and Acellular Pertussis Vaccine (Tdap) use in pregnancy as a regional priority aligned with Immunization Agenda 2030 (IA 2030), to reduce the burden of disease in younger infants (12).

## Public health emergency management

EMPHNET plays a critical role in managing health emergencies in the EMR, which faces persistent challenges such as conflict, natural disasters, and communicable diseases, further exacerbated by fragile health systems. Since its inception in 2017, the Public Health Emergency Management Center (PHEMC) at EMPHNET has supported priority countries by training Frontline Response Teams (FRTPs), and Rapid Response Teams (RRTs) on key public health topics while fostering robust collaborations with ministries and other partners.

Leveraging its access to remote areas and local-level expertise, PHEMC prioritizes strategic, sustainable approaches over *ad-hoc* interventions. Although outbreaks are often inevitable, PHEMC's alignment with national policies ensures a comprehensive approach to emergency management that covers preparedness, prevention, response, and recovery. PHEMC supports countries in establishing, operationalizing, and evaluating their public health rapid response systems by integrating principles, processes, and procedures into their existing structures through the co-development of guidelines and standard operating procedures (SOPs).

PHEMC adopts a country-focused approach, enhancing national and community preparedness and response efforts tailored to each country's specific needs. Many countries conducting Joint External Evaluation (JEE) exercises encounter challenges in developing practical National Action Plans for Health Security (NAPHS) due to limited technical capacities and multisectoral coordination. PHEMC enhances preparedness by building capacities through simulation exercises, RRTs, After Action Reviews, JEEs, and response capacity assessments. It also supports health at points of entry and offers technical assistance for developing national plans, guidelines, and operating procedures.

PHEMC strengthens emergency response capabilities at both national and regional levels by supporting Emergency Operations Centers, outbreak investigations, surge capacity, and multisectoral collaboration. These efforts include providing emergency assistance and facilitating integrated responses across sectors. The center supports data-driven emergency management through surveillance, early warning systems, and innovations in risk assessments, infodemic management, and Incident Management Systems. PHEMC promotes operational research, monitoring, evaluation, and the establishment of emergency rosters and communities of practice to inform decision-making throughout the emergency cycle. PHEMC also supports EMPHNET's operations and country-level logistics by enhancing stockpiling, procurement, and stock management mechanisms to ensure timely and effective emergency responses.

In addition, PHEMC integrates the One Health approach into all emergency management activities to address the interconnectedness of human, animal, and environmental health. It assists countries in strengthening emergency response plans by updating SOPs, continuity plans, and risk communication strategies. It conducts hazard assessments and risk mapping, such as for Chikungunya and floods in Sudan, and supports national planning efforts like Pakistan's 2023–2025 roadmap.

PHEMC bolsters border health strategies to mitigate cross-border disease transmission, aligning efforts with International Health Regulations. Collaborations include developing technical guidance for Libya, organizing regional symposia, and building the capacity of port health staff in Morocco to respond to public health events. Recognizing the health security challenges posed by mass gatherings, such as Iraq's Arba'een pilgrimage, PHEMC enhances surveillance, environmental monitoring, and response capacities. Activities include real-time infectious disease tracking, rapid responder training, and public health studies during these events. PHEMC addresses gaps in emergency risk communication by developing frameworks and strategies for outbreak preparedness. It supports Risk Communication and Community Engagement capacity building, rumor control, and social listening projects, improving responses during Coronavirus disease 2019 (COVID-19) and other emergencies.

Moreover, PHEMC promotes multisectoral coordination (MSC) principles, collaborating with partners including the United Kingdom Health Security Agency to implement frameworks tailored to country contexts. Efforts include raising awareness and translating MSC theory into practice through case studies and capacity-building initiatives. PHEMC builds the country's capacity to develop and conduct simulation exercises that identify preparedness gaps and improve emergency plans. These exercises are integrated into training for RRTs in Egypt, Jordan, Iraq, and Pakistan. Additionally, PHEMC supports countries in developing electronic databases, real-time dashboards, and operational research capacities. It facilitates data-driven decision-making and promotes research publication and policy brief development to guide emergency preparedness and response strategies.

## Health security, laboratory capacity, and biosecurity

EMPHNET has played a significant role in advancing biosafety and biosecurity in laboratories across the region. This includes the development of SOPs for handling hazardous materials and managing chemical waste to improve biosafety practices (13, 14). In Libya, EMPHNET collaborated with Georgetown University's Center for Global Health Science and Security (CGHSS) on a project to formalize biohazardous waste management in key health facilities. The project engaged stakeholders and provided technical support to prevent the misuse of biological materials and enhance biosafety and biosecurity practices. Similarly, in Iraq, EMPHNET partnered with the Ministry of Health to improve biosecurity in liberated areas, focusing on biomedical waste treatment, disposal, and knowledge sharing to strengthen biosecurity systems.

In Afghanistan, EMPHNET worked with the Ministry of Public Health to improve the handling and security of biological samples and the disposal of expired reagents. EMPHNET also focused on enhancing biosecurity in Jordan by establishing inventory systems for dangerous pathogens and collaborating with the Middle East Scientific Institute for Security (MESIS) to train laboratory technicians in Jordan and Morocco on the safe handling of biological toxins like Ricin and Botulinum (15).

In collaboration with Georgetown University's CGHSS, EMPHNET strengthened cross-border networks for the surveillance, detection, and response to potentially weaponizable pathogens, contributing to regional health security (16). Technical documents, policy briefs, and health situation updates were produced to guide public health actions and align regional health policies with global standards, thus bolstering the resilience of health systems (17).

In Jordan, EMPHNET and MESIS supported the National Biosecurity Committee by conducting a comprehensive situational analysis and risk assessment. This included reviewing legislation and standards and engaging stakeholders to identify and prioritize biosecurity needs (18). Furthermore, EMPHNET activated (RRTs in high-risk Iraqi governorates to enhance local capacity for emergency response and preparedness (19, 20).

Efforts to address transboundary zoonotic diseases in countries like Libya and Tunisia have improved regional coordination and information sharing, crucial for disease control and prevention. Additionally, a consortium of laboratories was established in Tunisia, Libya, and Morocco in partnership with CGHSS to manage high-consequence pathogens and ensure sustainable biosafety measures (21). These initiatives reflect EMPHNET's commitment to advancing health security, improving laboratory capacity, and fostering regional collaboration in the EMR.

## Neglected tropical diseases

EMPHNET has been instrumental in combating NTDs, particularly onchocerciasis in Yemen, the only Asian country endemic to onchocerciasis. In 2021, EMPHNET led a door-to-door Mass Drug Administration campaign across 29 districts, achieving 91% coverage of over 450,000 individuals despite conflict and logistical challenges (22). This success was driven by comprehensive training for health workers, volunteers, and teachers, enhancing community engagement. In 2023, EMPHNET shifted to a facility-based distribution model due to policy changes, covering 82% of the targeted population through 340 health facilities (23). A subsequent Coverage Evaluation Survey highlighted key areas for improvement, such as communication strategies, logistical support, and targeted interventions for underserved demographics.

EMPHNET's efforts have been bolstered by partnerships with organizations like the End Neglected Diseases (END) Fund, Task Force for Global Health, and Global Institute for Disease Elimination (GLIDE), enabling resource mobilization and alignment with national health priorities. By building local capacity, adapting to operational challenges, and fostering collaborations, EMPHNET has significantly contributed to onchocerciasis elimination efforts, emphasizing its vital role in advancing public health in the EMR.

## One health

The increasing rate of emerging infectious diseases, now rising at an estimated 6.7% annually, with up to 75% originating in animals, highlights the urgency of this approach (24). EMPHNET has prioritized the development of practical guides to support One Health implementation in the EMR. Its 2022 guide “Toward the Implementation of the One Health Approach in the EMR” provides actionable recommendations for establishing governance mechanisms, strengthening workforce capacity, and integrating environmental health considerations (25). Building on this, EMPHNET introduced a 2023 framework focused on addressing climate-related health risks, such as heat stress and water scarcity, through resilient public health policies tailored to the region's vulnerabilities (26).

A skilled workforce is critical for operationalizing One Health (27). To address this, EMPHNET introduced the “Regional Curriculum Framework for One Health Professional Training Program” offering flexible, multidisciplinary training tailored to regional needs. This initiative complements EMPHNET's integration of One Health principles into FETPs, which include mentorship workshops and cross-sectoral collaboration exercises. EMPHNET has also conducted Rapid Response Team RRT training across several countries to build local capacities in managing zoonotic outbreaks such as H5N1 and MERS-CoV.

Zoonotic diseases are a focal area for EMPHNET, given their impact on public and animal health. EMPHNET's initiatives include training programs for Anthrax surveillance in Jordan, Iraq, and Bangladesh, improving early detection and outbreak response capabilities (28). Similarly, its projects addressing Brucellosis in Jordan, Pakistan, and Morocco have enhanced diagnostic capacity and surveillance infrastructure. Between 2018 and 2022, EMPHNET trained over 150 clinicians in Jordan, conducted investigations of animal farms, and studied risk factors among vulnerable populations such as Syrian refugees (10, 29). These efforts also extend to vector-borne diseases like Crimean-Congo Hemorrhagic Fever, where EMPHNET has supported tick surveillance and diagnostic improvements in Afghanistan. EMPHNET collaborated with regional and global partners in 2023 to strengthen frameworks for managing Transboundary Zoonotic Diseases through system mapping and strategic recommendations (30).

Recognizing AMR as a growing global threat, EMPHNET has implemented a project “Partnership for AMR Surveillance Excellence” (PARSE), which standardizes surveillance protocols and assesses capacities across multiple regions (31). Collaborative efforts in Bangladesh aim to understand AMR dynamics at the human-animal-environment interface, generating valuable data for intervention strategies.

Environmental factors significantly influence health outcomes, and EMPHNET has incorporated environmental health into its One Health agenda. Through projects such as environmental surveillance for *Burkholderia pseudomallei* in Bangladesh (32), EMPHNET has expanded the understanding of ecological determinants of health. These efforts align with its broader strategy to integrate climate resilience and environmental considerations into regional health planning.

## Non-communicable diseases

The EMR faces the highest rate of premature mortality due to the four main NCDs—cardiovascular diseases, cancer, diabetes, and chronic respiratory diseases (8). Since its inception, EMPHNET has played a transformative role in combating NCDs across the EMR. By fostering innovation, collaboration, and evidence-based approaches, EMPHNET has established itself as a leader in NCD prevention and control. One of its flagship initiatives is the establishment of the Eastern Mediterranean NCD Research and Prevention Center, launched in 2023. This center connects regional priorities with actionable interventions, guided by EMPHNET's comprehensive NCD Operational Guide. The framework emphasizes NCD surveillance, prevention, management, and the integration of digital technologies to enhance healthcare delivery (33).

EMPHNET's commitment to capacity building is evident in its innovative approaches, such as the (34). Launched on World Field Epidemiology Day in 2023, these grants support projects that address NCD gaps, engaging FETP residents and public health professionals (35). Complementing these efforts is the NCD Implementation Research Toolkit, designed to guide the effective application of implementation science. This e-learning resource, freely available through EMPHNET's Learning Management System, enables professionals to enhance their knowledge and skills flexibly and efficiently (36).

Recognizing the importance of systemic planning, EMPHNET collaborated with global partners to develop the Non-communicable Disease Capacity Assessment and Planning (N-CAP) Process (37, 38). This initiative supports ministries of health in evaluating and prioritizing public health strategies to combat NCDs. Implementations in Jordan, Iraq, and Pakistan have enabled stakeholders to assess healthcare functions comprehensively and devise strategic plans tailored to their contexts (39).

EMPHNET also prioritizes the integration of NCD services into primary healthcare. Through the Family Health Teams approach, the organization collaborates with countries like Jordan to modernize primary care systems (40, 41). These efforts have improved access to quality healthcare for vulnerable populations, including refugees, while reducing financial burdens associated with out-of-pocket expenditures.

Additionally, EMPHNET's public health campaigns, such as the “United Against Tobacco and COVID” initiative, have had a profound impact (42, 43). Reaching over 50 million people across the region, the campaign raised awareness about tobacco harms, influenced policy changes, and promoted smoke-free environments (44). Similar campaigns addressing hypertension, salt reduction, and cervical cancer have demonstrated measurable improvements in public health behaviors and outcomes.

EMPHNET's commitment to advancing evidence-based solutions is further reflected in its use of implementation research. For instance, the organization evaluated the Integrated NCD-Humanitarian Response project in Jordan to ensure its scalability and sustainability. The implementation research not only improved service uptake but also integrated NCD interventions into routine healthcare practices (45). EMPHNET also joined the Jordan Ministry of Health in 2019 in adapting, implementing, and evaluating the WHO's HEARTS technical package in primary healthcare centers in Jordan (46, 47).

EMPHNET has made significant strides in addressing mental health challenges in the region, including building the capacity of frontline health workers to conduct mental health screening and diagnosis (48, 49).

The significance of EMPHNET's contributions was recognized in 2022 when it received the United Nations Interagency Task Force and WHO Special Programme on Primary Healthcare Award (50). This honor underscores EMPHNET's pivotal role in advancing multisectoral coordination and improving NCD care across the EMR.

## Women and child health

EMPHNET is addressing the significant healthcare needs of women and children in the region, focusing on high mortality rates in several countries, challenges faced by women in conflict and fragile settings, and issues such as neonatal deaths. The organization addresses these needs through strengthening health systems to ensure sustainable health services for all, with an emphasis on Primary Health Care (PHC). EMPHNET supports innovative PHC models, like the Family Health Approach, to improve resource mobilization and ensure the equity, quality, and accessibility of health services.

EMPHNET focuses on strengthening surveillance and information systems for maternal and child health. Notable initiatives include establishing a harmonized, digital Reproductive Health Registry (hRHR) in Mafraq, Jordan, providing technical assistance for the Jordan Maternal Mortality Surveillance and Response (JMMSR) System, and supporting neonatal mortality audits in the Azraq and Zaatari refugee camps. To ensure that interventions meet specific needs, EMPHNET involves women in the design and delivery of services. This approach was used in creating a low-intensity psychosocial intervention package for Syrian adolescent females and in conducting comprehensive qualitative research on community views of family planning accessibility for Syrian refugees and vulnerable host communities, which informs policy and strategy development, as well as efforts to digitize family planning services.

In addition, EMPHNET has undertaken projects to strengthen community engagement. This community engagement extended to health promotion campaigns, including a large-scale campaign against cervical cancer and the Integrated School Health project, which involved the local organizations and the community to ensure comprehensive health services for Syrian refugee children in both formal and informal education settings (51).

EMPHNET also prioritizes generating evidence on women's health through field investigations, research projects, surveys, assessments, and original studies, many of which are published in peer-reviewed journals. These initiatives aim to provide actionable insights, contributing to the body of knowledge on women's health and supporting informed decision-making and intervention design.

## Public health surveillance and digital health

EMPHNET has significantly contributed to strengthening public health surveillance systems in the EMR. A key component of this work is its support for FETPs, which are hosted within ministries of health to train epidemiologists. These programs focus on enhancing surveillance and response capabilities through hands-on training, case studies, and real-world assessments.

Maternal and neonatal mortality surveillance is another critical focus area for EMPHNET. In 2024, EMPHNET collaborated with Pakistan to develop its Mortality Surveillance System to enhance tracking and response efforts. Similarly, between 2016 and 2020, EMPHNET supported the implementation of the JMMSR system, which trained over 1,040 health workers and improved data collection and analysis for better maternal health outcomes.

EMPHNET has also worked with the United Nations High Commissioner for Refugees (UNHCR) to conduct neonatal mortality and stillbirth audits among Syrian refugees in camps like Za'atari and Azraq. These audits identify risk factors and gaps in care quality to guide improvements. The findings are shared with stakeholders for collaborative problem-solving.

In Iraq, EMPHNET has supported real-time syndromic surveillance during the Arba'een mass gatherings since 2014. These efforts provide actionable data to manage health risks associated with large-scale events. Similarly, in Somalia, EMPHNET addressed gaps in vaccination and outbreak response by improving surveillance data management for polio and measles between 2022 and 2023.

Globally, EMPHNET participated in the PARSE project, which focused on AMR in low-income countries. Supported by the Fleming Fund UK, this initiative developed SOPs and mapped capacities in 16 countries (52). EMPHNET also conducted a bibliometric analysis of public health surveillance literature in the EMR to highlight research gaps and inform future strategies (6).

EMPHNET has been a strong advocate for integrating digital health and e-registries into public health systems. One notable project is the *h*RHR in Jordan, which connects maternal and child health data across healthcare sectors. Funded by the International Development Research Centre (IDRC), this initiative improved data access and supported better decision-making.

Another initiative focused on improving family planning services for Syrian refugees and host communities in Jordan and Lebanon. Using mobile technology and culturally sensitive counseling, the project encouraged contraceptive use and enhanced the quality of care. It was implemented in collaboration with local ministries and academic institutions.

During the COVID-19 pandemic, EMPHNET played a key role in developing Jordan's Ministry of Health COVID-19 dashboard, which provided real-time data to guide public health responses. Additionally, EMPHNET contributed to drafting Jordan's National Digital Health Strategy (2023–2030) and aligning it with WHO's Global Strategy on Digital Health.

To further integrate digital health into public health, EMPHNET developed an operational guide for the EMR to enhance service delivery and management through digital tools, ultimately improving population health outcomes.

## Public health workforce capacity

EMPHNET has contributed to strengthening the public health workforce in the EMR by conducting training programs, enhancing skills, and supporting public health programs (53). These efforts are crucial for improving the capacity of countries to manage public health threats. The Workforce Capacity (WFC) unit, under EMPHNET's Center of Excellence for Applied Epidemiology (CEAE), offers customized training in applied epidemiology, emergency preparedness, immunization programs, and other public health areas. These training programs are delivered through face-to-face, online, and blended learning formats to suit regional needs and learner preferences.

EMPHNET's eLearning Unit, established to meet the growing demand for online learning, provides free eLearning resources for public health professionals. The EMPHNET Learning Management System (LMS) hosts more than 130 h of self-paced learning and offers blended FETP courses. These online programs are designed to accommodate diverse needs and geographic locations, ensuring access to quality public health education across the region. The E-Learning Unit also supports partners in establishing and enhancing Learning Management Systems.

FETPs are key to enhancing the epidemiologic capacity of public health workforces in the EMR. These programs focus on building practical field epidemiology skills necessary for preventing, detecting, and responding to health threats. EMPHNET plays a pivotal role in supporting and expanding FETPs across the region, ensuring that these programs are integrated into national health systems. The programs have successfully produced a significant number of graduates, contributing to enhanced public health response capacity. To date, EMPHNET has supported over 15 countries and has implemented more than 35 FETP tiers, with over 200 cohorts and 3,000 graduates (54). EMPHNET works with countries to institutionalize these programs within national health systems, ensuring they remain aligned with national priorities and global health standards.

EMPHNET works on enhancing scientific writing skills by encouraging and supporting the production of scientific publications by public health workers and experts (55). These publications contribute to the global public health knowledge base and highlight the achievements and findings of FETPs in the EMR through different online courses and national and regional workshops. EMPHNET applies the Kirkpatrick Evaluation Model to assess the long-term effectiveness of its training programs. Additionally, the WFC helps mobilize resources during public health emergencies by deploying trained professionals to respond to outbreaks and disasters. The response capacity is evident in EMPHNET's contributions to managing disease outbreaks and assisting with conflict, natural or man-made disaster relief efforts, and other crises in the EMR that affect the population's health.

## Public health research

Research and evidence generation are central to EMPHNET's mission. Collaborating with academic and public health institutions, EMPHNET conducts operational research on diverse topics. These efforts have resulted in numerous peer-reviewed publications, policy briefs, and teaching case studies. Capacity-building initiatives have empowered public health professionals in research methods, scientific writing, and the use of statistical software (55–58).

Self-paced online courses on qualitative research, public health ethics, and citation management tools like Mendeley have further expanded access to knowledge. EMPHNET has also supported researchers through operational research mini-grants and COVID-19 response funding.

## Media, communication, and networking efforts for advancing public health

EMPHNET plays a pivotal role in advancing public health in the EMR through strategic media, communication, and networking efforts that disseminate knowledge and foster collaboration. Operating in a region marked by sociopolitical unrest, natural disasters, and limited resources, EMPHNET addresses critical barriers to information exchange and professional development with innovative approaches.

The organization has established platforms to facilitate knowledge sharing, including its Webinar Series, (59) which began during the COVID-19 pandemic to combat misinformation. This series has since expanded to cover broader public health topics, featuring 44 webinars and 180 speakers, and engaging over 6,000 participants. EMPHNET also organizes biennial regional conferences, attracting hundreds of participants for networking, policy discussions, and knowledge dissemination through presentations, workshops, and publications (60).

To enhance visibility and communication, EMPHNET produces a range of publications, such as EMPHNET Connect, Research Digest, Emergency Bulletin, and the FETP Newsletter. These, along with social media outreach, website content, and multimedia materials, share success stories, research updates, and public health opportunities. The organization's EpiNews platform, accessible via a portal and mobile app, further provides timely updates on public health developments in the region (61).

EMPHNET invests heavily in capacity building through initiatives like the Engage Internship Program. Launched in 2019, this program has provided over 80 interns with hands-on public health experiences, earning accreditation from the Association of Schools of Public Health in the European Region (APHEA) and resulting in employment opportunities for many participants. The program's achievements are highlighted in the Engage Newsletter (62). Furthermore, EMPHNET collaborates with 17 FETP Ambassadors who promote FETPs by sharing program achievements, leading to the publication of hundreds of news articles, social media posts, and newsletters (63).

The organization enhances resource accessibility through its EMPHNET Electronic Library (EEL), a free subscription-based platform offering gray literature and research materials to over 750 subscribers, benefiting students, professionals, and FETP residents (64). EMPHNET also champions health advocacy and awareness through its media channels, running impactful campaigns such as the 2023 Cervical Cancer Campaign, which reached over two million women in Jordan.

## Key challenges facing EMPHNET

EMPHNET operates in a region where persistent and multifaceted challenges frequently hinder its ability to deliver public health programs effectively. Political instability and conflict remain among the most critical barriers, with more than half of the EMR countries experiencing acute or protracted emergencies. Such conditions create volatile environments marked by violence, displacement, and disrupted governance, making it exceptionally difficult to implement programs such as vaccination campaigns or disease surveillance and requiring complex logistical and security arrangements.

Compounding these difficulties are fragile health systems with weak infrastructure, severe shortages of trained public health professionals, and limited operational capacity. These systemic gaps slow disease detection and response, while the recruitment, training, and retention of skilled personnel remain ongoing struggles. Sustaining gains in such contexts is particularly challenging without reliable and continuous support.

A central and recurring constraint is funding. Limited and often short-term funding not only restricts the scale and scope of interventions but also hampers long-term planning and sustainability. In crisis-affected areas, these funding limitations are further compounded by high operational costs, including those related to transportation, security, and supply chain management.

Resource and logistical challenges—such as accessing remote communities, managing disrupted supply lines, and overcoming shortages of essential supplies—further delay or restrict program implementation. While EMPHNET has developed adaptive strategies, including close collaboration with local partners, flexible delivery models, and targeted capacity-building initiatives, the combination of unstable political contexts, systemic health weaknesses, and funding constraints continues to pose significant obstacles.

## Key lessons learned by EMPHNET

Through years of operating in complex and often unstable environments, EMPHNET has learned that strong partnerships and networks are essential for success. Collaboration with ministries of health, international organizations, academic institutions, and communities amplifies impact and enables more effective responses to public health threats. Maintaining a strong regional presence and tailoring interventions to each country's cultural, social, and political context have proven critical for achieving results, as has investing in local capacity to ensure sustainability. Continuous monitoring and evaluation allow EMPHNET to identify what works, make timely adjustments, and maintain accountability. Flexibility and innovation—such as adopting alternative delivery models, using digital surveillance, or applying One Health approaches—have been vital for sustaining operations during crises. Above all, EMPHNET's experience demonstrates that public health networks play a pivotal role in building resilience, strengthening preparedness, and fostering a sense of shared responsibility across borders. These lessons continue to shape the organization's strategies for advancing public health in the EMR.

## Implications of the findings for public health policy in the EMR

EMPHNET's experience highlights several important directions for public health policy in the EMR. First, regional and national health strategies should formally integrate and support collaborative networks like EMPHNET, recognizing their value in enhancing resilience, preparedness, and cross-country coordination. Aligning national policies with global health security frameworks can ensure that local efforts contribute to global goals while benefiting from shared expertise. Strengthening the public health workforce must remain a policy priority, with sustained investment in training programs such as FETPs and continuous professional development for epidemiologists, laboratorians, and other specialists. Effective responses to complex health challenges require enabling multi-sectoral and cross-border collaboration, supported by data sharing, joint training, and mutual assistance agreements. Finally, policies should be evidence-driven and adaptable to innovation, ensuring that proven interventions, new technologies, and operational research findings are rapidly institutionalized. Overall, EMPHNET's case demonstrates that embedding networks into the health architecture, prioritizing workforce capacity, and fostering coordinated, forward-looking policy frameworks can significantly advance health outcomes and preparedness in the EMR.

## Implications of the findings for future public health research in the EMR

The findings also point to clear priorities for future research in the region. Operational and implementation research should be embedded in public health practice, with studies designed in partnership with programs to address real-world challenges and inform policy. Building regional research capacity—through training, data systems, and coordinated research networks—will enable multicountry studies on shared health threats and enhance evidence-based decision-making. Addressing identified knowledge gaps, such as health system strengthening in fragile settings, neglected endemic diseases, mental health, climate change impacts, and AMR, is essential for meeting emerging health needs. Finally, the effectiveness and sustainability of network-based interventions themselves warrant study, including their cost-effectiveness, governance, and role in driving health policy and innovation. By pursuing action-oriented, collaborative, and priority-driven research, the EMR can generate the evidence needed to continually improve its public health systems and outcomes.

## Conclusion

The EMR faces a multitude of public health challenges, compounded by endemic diseases, recurrent outbreaks, and fragile health systems. Through its strategic initiatives, such as enhancing disease surveillance, supporting immunization campaigns, and strengthening workforce capacity, EMPHNET has been instrumental in improving health outcomes in the region. Additionally, its work in areas like maternal and child health, digital health innovations, and AMR highlights the network's comprehensive approach to strengthening public health systems. The collaboration between EMPHNET and various stakeholders, including ministries of health, international organizations, and academic institutions, has proven essential for addressing both regional and global health threats.

## Data Availability

The original contributions presented in the study are included in the article/supplementary material, further inquiries can be directed to the corresponding author.
